# Design and Development of an Imitation Detection System for Human Action Recognition Using Deep Learning

**DOI:** 10.3390/s23249889

**Published:** 2023-12-18

**Authors:** Noura Alhakbani, Maha Alghamdi, Abeer Al-Nafjan

**Affiliations:** 1 Information Technology Department, College of Computer and Information Sciences, King Saud University, Riyadh 11543, Saudi Arabia; nhakbani@ksu.edu.sa (N.A.); 443204753@student.ksu.edu.sa (M.A.); 2 Computer Science Department, College of Computer and Information Sciences, Imam Mohammad Ibn Saud Islamic University, Riyadh 11432, Saudi Arabia

**Keywords:** human action recognition, imitation detection, deep learning, transfer learning

## Abstract

Human action recognition (HAR) is a rapidly growing field with numerous applications in various domains. HAR involves the development of algorithms and techniques to automatically identify and classify human actions from video data. Accurate recognition of human actions has significant implications in fields such as surveillance and sports analysis and in the health care domain. This paper presents a study on the design and development of an imitation detection system using an HAR algorithm based on deep learning. This study explores the use of deep learning models, such as a single-frame convolutional neural network (CNN) and pretrained VGG-16, for the accurate classification of human actions. The proposed models were evaluated using a benchmark dataset, KTH. The performance of these models was compared with that of classical classifiers, including K-Nearest Neighbors, Support Vector Machine, and Random Forest. The results showed that the VGG-16 model achieved higher accuracy than the single-frame CNN, with a 98% accuracy rate.

## 1. Introduction

Human action recognition (HAR) involves the identification and classification of human actions from digital video data. It is a crucial area of research in computer vision, machine learning (ML), and Artificial Intelligence (AI) [1,2]. It has several applications across different domains, including sports analysis, surveillance and security, military, health care, robotics, and human–computer interaction [3].

AI and ML have facilitated the development of HAR systems by enabling computers to learn from large datasets of human actions. Deep learning (DL) algorithms, such as convolutional neural networks (CNNs) and recurrent neural networks (RNNs), have been used to automatically extract features from video data and accurately classify human actions. Recently, there have been notable advancements in deep learning approaches within the field. For instance, Xiong et al. proposed a deeply supervised subspace learning technique for assisting robots in comprehending object characteristics without direct contact [4]. In [5], a novel concept for learning feature interaction in cross-spectral image patch matching was introduced, resulting in the development of a new feature interaction learning module. Shu et al. in [6] presented the Omni-Training framework, which connects pretraining and metatraining for effective few-shot learning with limited data. These algorithms can learn complex patterns and relationships among different features of human actions, making them more effective in recognizing and classifying actions than traditional ML approaches [7].

In recent years, there has been a significant increase in the number of studies and publications and in the amount of research on HAR systems. Several research methodologies have been used in these studies, resulting in a diverse range of outcomes. The results obtained depended on various factors, such as the quality of the dataset, the recording protocol, data preprocessing techniques, the type of features detected, the number of participants involved, and the intended applications of the HAR system [1,2].

Although there have been a significant number of design studies published in this field, it is important to consider not only the technical aspects of the system but also its design implications. Addressing the challenges associated with the development of more accurate and effective HAR systems is crucial. These challenges include the variability and complexity of human actions, the need for a large number of labeled data, the ability to operate in real-world scenarios, the need to develop systems that are robust to individual differences, and ethical considerations. By addressing these challenges, we can develop HAR systems that are not only technically sound but also practical and ethical [3].

One specific application where HAR can be developed is in behavior recognition for children with Autism Spectrum Disorder (ASD). The detection and understanding of actions in children with ASD are crucial for early intervention and personalized therapy. However, the behavior of children with ASD presents unique challenges in capturing and interpreting, necessitating tailored solutions and specific datasets for accurate recognition.

In this study, we aimed to design and develop an imitation detection system by implementing an HAR algorithm using DL. Imitation tasks have significant applications in various fields, including autism therapy [8], robotics and games [9], and sports and motor education [10]. Initially, we developed a DL model that uses a single-frame CNN. Then, we used the transfer learning technique by implementing the pretrained VGG-16 model. We also compared their performance with that of different classical classifiers, such as K-Nearest Neighbors (KNN), Support Vector Machine (SVM), and Random Forest (RF). We evaluated our proposed models and compared them with those of previous studies using the same dataset.

This paper is structured as follows: Section 2 provides the background information, while Section 3 discusses related works. Section 4 and Section 5 elaborate on the research methods and the design considerations of the HAR framework. The experiment implementation is presented in Section 6, followed by the discussion of the system’s performance and evaluation in Section 7. Section 8 focuses on the results and a comparison of classical classifiers with related studies. Lastly, Section 9 concludes the paper and highlights future work.

## 2. Background

In this section, we provide the necessary a theoretical background about DL, and neutral networks, and HAR.

### 2.1. Human Action Recognition

In the last decade, HAR has emerged as an active research area in computer vision [1]. Herath et al. [11] define action as “the most elementary human-surrounding interaction with a meaning.” HAR is the process of labeling human actions within a given sequence of images, which becomes the classification of a human agent’s goals in a series of image frames [2]. Typically, the goal of action recognition is to discover the class of short, segmented, atomic actions [2]. In general, HAR is a hierarchical process, with lower levels focusing on human detection and segmentation.

The goal of those levels is to identify the regions of interest in a video that corresponds to static or moving humans. At the next level, visual information about actions is extracted and represented by features. Then, these features are used to recognize actions. As a result, recognizing an action based on features can be viewed as a classification problem [7].

Human action categorization remains a difficult task in computer vision. According to Vrigkas et al. in [12], HAR methods are categorized into two main categories, unimodal and multimodal, according to the nature of the sensor data they use. Methods for identifying human activities from the data of a single modality are known as unimodal human activity recognition methods. Most current models represent human actions as a set of visual features extracted from video streams or still images, and several classification models are used to recognize the underlying action. On the other hand, multimodal methods combine features collected from different sources. An event can be described using various features that provide additional and useful information. Several multimodal methods in this context are based on feature fusion, which can be expressed in two ways: early and late. The simplest way to take advantage of multiple features is to combine them in a larger feature vector and then learn the underlying action.

### 2.2. Deep Learning and Neural Networks

DL is a class of ML techniques that use many layers of information processing stages in hierarchical architectures for unsupervised feature learning and pattern classification. It is at the crossroads of neural network, graphical modeling, optimization, pattern recognition, and signal processing research [13]. DL has been demonstrated to be successful in various applications, such as computer vision, phonetic recognition, speech and image feature coding, handwriting recognition, and robotics, since 2006 [13].

DL networks are artificial neural networks with more than one hidden layer; therefore, DL networks are also known as deep neural networks [14]. The architecture of biological neurons, such as those of the human brain, inspired the design of an artificial neural network. The human brain consists of a massive network of interconnected neurons. Each neuron is a single cell that performs a specific task, such as responding to an input signal. Furthermore, when neurons are connected in a network, they can perform complex tasks, like speech and image recognition, with incredible speed and accuracy [15].

An artificial neural network is an interconnected group of nodes, with each circular node representing an artificial neuron and an arrow representing a connection from the output of one neuron to the input of another. An artificial neural network is made up of three layers: input, hidden, and output layers. The hidden layer connects the input and output layers [13].

The most established algorithm among various DL models is CNN, a type of artificial neural network that has been a powerful method in computer vision tasks [16]. The CNN is a type of multilayer neural network that is specifically designed to work with two-dimensional data, such as images and videos. The CNN is a mathematical construct composed of three types of layers (or components): convolutional, pooling, and fully connected layers. The first two layers, convolution and pooling, extract features, whereas the third layer, a fully connected layer, maps the extracted features to final output, such as classification [13].

## 3. Related Work

This section presents the related research that has been conducted in HAR. Table 1 shows a summary of HAR-related work.

Jaouedi et al. [17] proposed a novel method for using motion tracking, as well as human tracking, to extract spatial features from a video sequence. They used the Gaussian mixture model and Kalman filter methods to identify and extract moving people, and a gated RNN (GRNN) to collect features in each frame and predict human action. They evaluated the method on the University of Central Florida (UCF) Sports Action, UCF101, and KTH human action datasets, where they discovered several actions in various contexts. The experimental findings show that the proposed method is a good algorithm for HAR problems, with an average of 96.3% when tested on the KTH dataset, 89.1% on UCF Sports, and 89.30% on UCF101.

Yeole et al. [18] presented a method for automatically recognizing human activities in video sequences captured in outdoor areas using a single large-view camera. The CNN-VGG-16 and single-frame CNN models were used. They demonstrated the techniques using real-world video data to automatically distinguish normal behaviors from suspicious ones in a playground setting from films of continuous performances of six different types of human–human interactions: handshakes, pointing, hugging, pushing, kicking, and punching. According to their results, the single-frame CNN model outperforms CNN-VGG-16. Extensive experiments on the UT-interaction dataset were conducted to evaluate the performance of the proposed technique. The experimental results demonstrate that the average accuracy achieved by the VGG-16 approach was 60%, while the CNN approach achieved an accuracy rate of 90.46%. These findings indicate that VGG-16 performed poorly when applied to raw video graphic datasets.

Li [19] proposed a recognition information processing system based on DL, specifically utilizing an LSTM RNN, for gathering and identifying human motion data. The system includes three layers and enables the collection, processing, recognition, storage, and display of human motion data. By incorporating the LSTM RNN, the system achieves improved recognition efficiency, simplifies the recognition process, and reduces data missing rates caused by dimension reduction. The authors trained the model using the Pamap2 dataset and evaluated its performance and application effectiveness with real motion state analysis. The results demonstrate that the LSTM RNN outperformed traditional algorithms in terms of accuracy (98%).

Khan et al. [1] proposed a DL-based design for HAR. The proposed design includes several steps, such as feature mapping, fusion, and selection. Two pretrained models, DenseNet201 and InceptionV3, are considered for the initial feature mapping step. Then, the extracted deep features are fused using the Serial-based Extended method. Then, the best features are chosen using Kurtosis-controlled Weighted KNN. Several supervised learning algorithms are used to classify the selected features. The authors used several datasets, including KTH, IXMAS, WVU, and Hollywood, to demonstrate the efficacy of the proposed design. The proposed design achieved accuracy rates of 99.3%, 97.4%, 99.8%, and 99.9% on these datasets, respectively, according to experimental results. Furthermore, when compared with the state of the art, the feature selection step performed better in terms of computational time.

Ma et al. [20] suggested a novel deep convolutional generation confrontation network to recognize human motion poses. This method uses a deep convolutional stacked hourglass network to precisely extract the location of key joint points on the image. The network’s generation and identification components are intended to encode the first (parent) and second (child) hierarchies and to show the spatial relationship of human body parts. The generator and discriminator are designed as two network parts that are linked to encode the possible relationship of appearance, the possibility of the existence of human body parts, and the relationship between each part of the body and its parental part coding. The key nodes of the human body model and general body posture can be identified more precisely in the image. The method was evaluated on various datasets, LSP, LIP, and MPII. In most cases, the proposed method produced better results than other comparison methods.

## 4. Imitation Detection System Design Consideration

We aim to design and develop an imitation detection system by implementing an HAR algorithm using a DL approach. DL has recently shown promising results in the field of computer vision; it creates models by simulating human brain processing at multiple layers [1].

One of the widely used models in HAR is CNN. A CNN is a type of neural network that can recognize and classify features from frames. A CNN consists of three layers, convolutional, pooling, and fully connected layers [18]. The convolutional layer serves as a local feature extractor, and the pooling layer combines semantically similar features into one feature. The last layer is a standard neural network working as a classifier (or a standard classifier, such as SVM) that identifies the output from feature extraction and predicts the class of the frames based on the features extracted in the earlier steps [18]. As a result, the network picks up a collection of useful attributes for a classifier [7].

**Figure 1:**
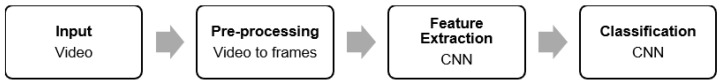
HAR algorithm.

Our system is based on a deep neural network to detect successful imitation in children. The algorithm employed is illustrated in Figure 1. Recordings serve as input, which undergoes video preprocessing to generate video frames. The CNN is then utilized to extract features from these frames, followed by classification. The CNN has emerged as a key algorithm for gesture detection in image classification due to its fast and stable classification capabilities. It can learn features without the need for manual extraction and train on raw images to generate feature extraction classifiers [21].

Conversely, for the training part, we aim to train the model using the KTH dataset [22], which is an action dataset containing six types of human actions (walking, jogging, running, boxing, hand waving, and hand clapping).

## 5. Materials and Methods

In this section, we will present the various stages involved in designing and implementing our clapping imitation detection system.

Section 5.1 will describe the dataset that was used for both training and testing. Then, we will describe the concept of transfer learning and how it is leveraged in our system in Section 5.2. Furthermore, we will provide a thorough explanation of the proposed system, including all the preprocessing steps required to prepare the dataset. We will also outline two different approaches to construct the model; then, we will present the evaluation of the two approaches.

### 5.1. Dataset

The goal of the imitation detection system is to detect the hand clapping action. So we used the KTH dataset [22], which is a video dataset that provides a collection of actions performed by humans. The actions comprise six different types, namely, walking, jogging, running, boxing, hand waving, and hand clapping.

**Figure 2:**
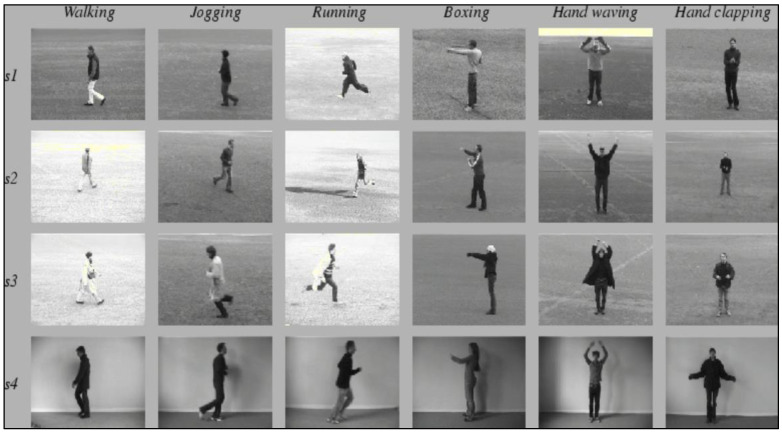
KTH dataset.

Each action was performed multiple times by a total of 25 subjects, across four different scenarios. The scenarios are characterized by the type of location where the actions take place, which are outdoors (s1), outdoors with scale variation (s2), outdoors with different clothes (s3), and indoors (s4) as illustrated in Figure 2.

All sequences were taken with a static camera operating at a 25-fps frame rate and against a homogenous background. The resulting sequences were uniformly downsampled to a spatial resolution of 160 × 120 pixels and have an average duration of 4 s. The video files are stored in AVI format, resulting in 600 video files for each combination of 25 subjects, 6 actions, and 4 scenarios.

We split the data into two categories for binary classification, where one is the “clapping” category, which includes hand clapping videos, and the rest of the videos fall into the “non-clapping” category. Table 2 shows a description summary for the KTH dataset.

Several research articles used this dataset; for example, Paramasivam et al. [23] created a novel solution to address the common obstacles encountered in existing approaches to HAR, which often use computationally intensive networks like 3D CNNs and two-stream networks. This involved developing HARNet, which is a lightweight directed acyclic graph-based residual 2D CNN with reduced parameters. HARNet was engineered from the ground up to overcome these challenges. The performance of the proposed method was assessed through experimentation using the KTH dataset.

Moreover, Zhang et al. [24] introduced a new video representation technique that improves motion recognition in videos by utilizing speeded-up robust features and two filters. They assessed the effectiveness of this video representation in action classification across various motion video datasets, including KTH. In comparison with existing methods, the proposed approach demonstrated superior performance on all datasets.

### 5.2. Transfer Learning

To train a new DL model from scratch, it is necessary to have a vast number of data, powerful computational resources, and numerous hours—or days—of training. Collecting and annotating domain-specific data can be a lengthy and costly process, making it difficult to apply DL models in real-world scenarios [25].

To address this problem, researchers have taken inspiration from the way the human visual system works. Humans learn multiple categories throughout their lives, from just a few examples. It is thought that this is possible because humans accumulate knowledge over time and use it when learning new objects. This approach can help overcome the need for a large number of data in DL models; this concept is transfer learning [25].

Transfer learning is the process of transferring and using knowledge obtained from a particular task to solve other related ones. The information used for transfer includes classifier weights, features, and data instances [26,27]. New techniques in transfer learning with DL have the objective of decreasing the amount of time and cost involved in the training process, as well as reducing the need for large datasets, which can be difficult to collect in certain sectors, like medical image analysis. Furthermore, developments in transfer learning with DL are paving the way for more powerful and intuitive AI systems, as it recognizes learning as a continuous process [28].

An untrained DL model is initialized with random weights for its nodes. Then, during the costly training process, these weights are optimized, using an optimization algorithm for a particular dataset, to achieve the best possible values. Interestingly, if the weights are initialized on the basis of a pretrained network, which may have been trained on quite different datasets, it can substantially improve the training performance in comparison with random weight initialization [28].

## 6. Proposed System

For detecting clapping imitation, we attempted two different approaches to implement our system. Initially, we developed a DL model that uses a single-frame CNN, which was given by Aruna et al. [29]. Moreover, we used the transfer learning technique by implementing the pretrained VGG-16 model. These methods are explained next.

### 6.1. Single-Frame CNN

**Figure 3:**
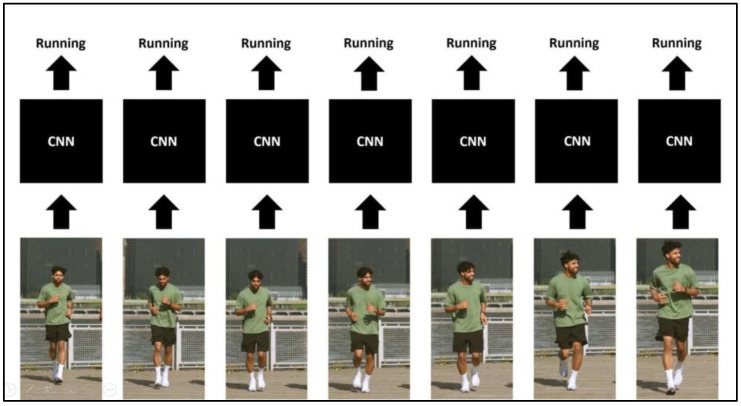
Single-frame CNN.

The single-frame CNN approach is designed to perform image classification on every single frame of a video to recognize the action being performed, as shown in Figure 3 [29]. In our case, it identifies if a frame contains a “clapping” or “non-clapping” action. The model generates a probability vector for each input video frame, which denotes the probability of the action being presented in that frame. Then, we average all the individual probabilities to obtain the final output probability vector [29]. The implementation of our approach involves three main steps, namely, preprocessing, feature extraction, and classification.

Preprocessing: First, we split the videos in the dataset into two sections; create two folders for each one, “clapping” and “non-clapping”; then store all the videos in these folders before reading each file individually; and extract the frames. To extract video frames from the class directories of the dataset, an iterative approach is followed. The first step is to loop through all the video files present in the dataset’s class directories, reading each video file using the OpenCV (“OpenCV is an open-source computer vision and machine learning software library” [30]) library, extracting the video frames, and then iterating through each video frame to resize it to a fixed size (64 × 64 pixels) and normalize the pixel values. The last step in this stage is to create the final preprocessed dataset by returning the preprocessed frames and their associated labels as NumPy (“NumPy is a Python library that provides a multidimensional array object and other mathematical computations” [31]) arrays.

**Figure 4:**
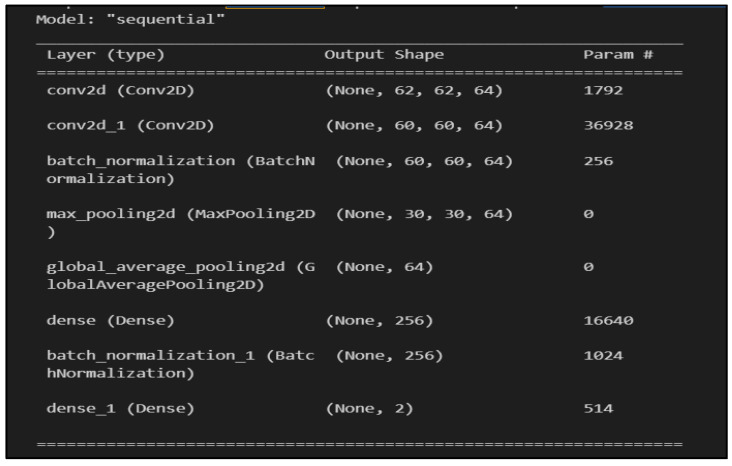
CNN classification model structurer.

Feature extraction and classification: As shown in Figure 4, the CNN classification model is structured with (1) two convolutional layers, (2) two batch normalization layers, (3) one max-pooling layer, (4) one global average pooling layer, and (5) two fully connected (dense) layers.

The first two convolutional layers apply 64 filters of size 3 × 3 each to the input image. These filters, also known as kernels, play a crucial role in creating a feature map for the subsequent layer [32]. Both layers use the rectified linear unit (ReLU) activation function, which is a piecewise linear function that returns the input if it is positive; otherwise, it outputs zero [32]. A batch normalization layer is included after the second convolutional layer, which contributes to the stabilization of the learning process by normalizing the output of the previous layer [33].

The max-pooling layer reduces the spatial dimensions of the output, and the global average pooling layer further reduces the dimensions by averaging the values in each feature map. A dense layer with 256 units and ReLU activation is added, followed by another batch normalization layer. The final dense layer uses the Sigmoid activation function to convert the output into a probability distribution ranging from 0 to 1.

After building the model, we define the function make_average_predictions(), which takes the video path and n frames. This function produces a singular prediction from an entire video. This function operates by selecting n frames from the entire video and making predictions based on those frames. Subsequently, the predictions of those n frames are averaged to determine the final action class name for the entire video. This process using the moving average formula is shown as follows [29]:

$$P_{f}=\frac{\sum_{i=n+1}^{0}P_{i}}{n}$$

where *n* is the number of frames to be averaged; *P_f_* is the final predicted probability; *P* is the probability predicted for the current frame; *P* − 1 is the probability predicted for the last frame; *P* − 2 is the probability predicted for the second-last frame; and *P* − *n* + 1 is the probability predicted for the (*n* − 1) last frame.

Finally, we run the single-frame CNN image classification model on each frame in a particular video and send the list of predictions to the moving average function to return the average of all class (clapping and non-clapping) probabilities to obtain a final probability vector. Then, the highest probability class is the predicted class for the entire video.

### 6.2. VGG-16

**Figure 5:**
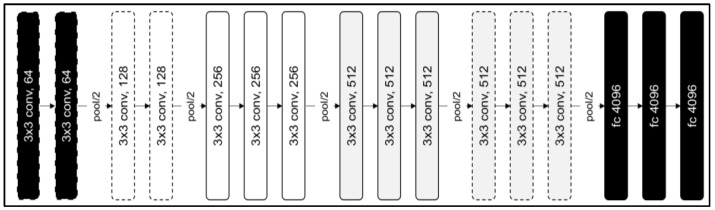
VGG-16 architecture.

The VGG-16 architecture, also known as Visual Geometry Group-16, is a DL model developed by University of Oxford researchers. VGG-16 is well known for its simplicity and low computational complexity when compared with other DL models [34]. The VGG-16 architecture is a CNN architecture broadly used for image classification. In general, the models based on VGG-16 have achieved high accuracy [35]. It is composed of 16 weight layers in total, including 13 convolutional layers, 3 fully connected layers, and 5 max-pooling layers [34], as shown in Figure 5.

The first layer of the network responsible for processing the input image is referred to as the input layer. Typically, the input image data are resized to a fixed dimension, such as 224 × 224 pixels, before being fed into the network. There are 13 convolutional layers in the VGG-16 model, which are responsible for extracting features from the input image using filters or kernels [34]. Notably, the VGG-16 model uses small 3 × 3 filters in its convolutional layers to enable the network to learn complex patterns with fewer parameters. An activation layer follows each convolutional layer and applies the ReLU activation function [34].

Additionally, the architecture contains five pooling layers that are intended to decrease the spatial dimensions of the feature maps to reduce computational complexity and control overfitting. VGG-16 uses max-pooling, which keeps the highest value within each pooling window. Finally, three fully connected layers combine the features extracted from the preceding layers to create final predictions. The last fully connected layer has a SoftMax activation function that produces probabilities for each class [34].

The VGG-16 pretrained CNN model is used in this work. We chose this architecture because it is well established and has demonstrated superior performance in the field of image classification [36].

Preprocessing: First, we split the videos in the dataset into two sections; create two folders for each one, “clapping” and “non-clapping”; then store all the videos in these folders before reading each file individually; and extract the frames using the OpenCV library. Then, each image is resized to a fixed size (224 × 224 pixels) using image interpolation in the OpenCV library, which is a conversion process from a low-resolution image to a high-resolution image by resizing and performing image zooming, enhancement, and reduction [37].

Data augmentation is a technique for increasing the sample set using techniques such as translation, horizontal flipping, and noise disturbance to reduce the “overfitting” of deep CNN caused by limited training samples [38]. After splitting the data into training and testing, we applied data augmentation on training data using the ImageDataGenerator class from the TensorFlow (“TensorFlow is an open-source software library for high-performance numerical computation” [39]) library.

**Figure 6:**
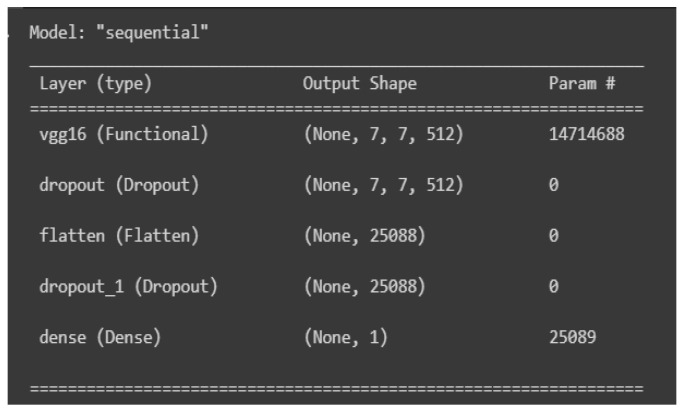
VGG-16 model structure.

Feature extraction and classification: We load the base model, which is VGG-16, to be the first layer; then, we add a dropout layer, which is a technique to prevent overfitting by randomly dropping out units in the neural network [40]. Then, we add a flatten layer to make the multidimensional input one-dimensional, and another dropout layer is added; finally, we add the dense layer using a sigmoid activation function to produce output values ranging from 0 to 1, because we want binary classification. A summary of the structure is shown in Figure 6. Furthermore, we set the VGG-16 model’s trainable parameter to False to freeze its weights.

## 7. Evaluation

Upon finishing the training process of our models and applying them to our data, we proceeded to evaluate their performance using various metrics. These performance measures include confusion matrix, accuracy, precision, recall, and F1. By comparing the performance of our models, we were able to gain a comprehensive understanding of their performance and identify the most efficient approach in generating accurate predictions.

We plotted the total loss vs. total validation loss graph and the total accuracy vs. total validation accuracy graph for both models, as shown in Table 3. We can see that the single-frame CNN in both graphs predicted the training set correctly, as denoted by its smooth line. However, in the validation set, the line goes up and down randomly, so we believe that this is an indication of overfitting.

Table 4 shows the obtained confusion matrix. In a confusion matrix, there are typically four categories: true positive, which represents positive instances that have been correctly identified; false positive, which refers to instances that are negative but have been incorrectly labeled as positive; true negative, which reflects negative instances that have been correctly identified; and finally, false negative, which indicates positive instances that have been incorrectly predicted as negative [33].

The single-frame CNN and VGG-16 models correctly classified 98% and 96% of the true-positive class, respectively, and for the true-negative class, the single-frame CNN and VGG-16 models correctly classified 85% and 100% predictions, respectively. Additionally, in terms of false positives, the single-frame CNN and VGG-16 models incorrectly classified 2.4% and 4% of the clapping labels as non-clapping, respectively. Finally, for the false negatives, the single-frame CNN and VGG-16 models incorrectly classified 15% and 0% of the non-clapping class as clapping, respectively.

The proposed models were compared with traditional ML models: RF, KNN, and SVM. The KNN classifier, among several classifiers available, relies on a distance or similarity function, such as the Euclidean distance, to evaluate pairs of observations. It is a case-based learning algorithm that utilizes this function to make classifications [41]. SVM is effective in handling datasets with a high number of dimensions.

In cases where the data are nonlinear and datasets cannot be easily separated, kernels are used to project the data into a higher-dimensional space, where it becomes feasible to separate the data linearly [41]. Finally, the RF technique is rooted in the idea of ensemble learning, where multiple classifiers are combined to tackle intricate problems and enhance model effectiveness. By extending the principles of decision trees, RF enables the construction of more robust models.

The evaluation of the performance metrics is shown in Table 5, which presents various measures—accuracy, precision, recall, and F1. Accuracy shows the ratio of total observations that have been predicted correctly. Precision represents the ratio of correctly predicted positive observations to total predicted observations that are positive. Recall denotes the ratio of correctly predicted positive observations to total observations in the actual class. Lastly, F1 is a weighted average of precision and recall [34].

The results show that the VGG-16 model achieved higher accuracy compared with the single-frame CNN model. In terms of precision, there is a minor difference between the two models, given that the single-frame CNN and VGG-16 models scored 97% and 96%, respectively. However, there is a notable difference between the two models with respect to recall, where the VGG-16 and single-frame CNN models attained 100% and 85% recall, respectively. Last, regarding the F1 measure, the VGG-16 and single-frame CNN models had rates of 98% and 90%, respectively.

## 8. Result and Discussion

In addition to the confusion matrix results, which show that the VGG-16 model outperformed the other method, the classification accuracy of our model was also compared to other previous studies that used similar approaches, where they used the same dataset but different extracted features classification techniques, as shown in Table 6.

For instance, David and Abbas, in [42], addressed the difficulties in capturing videos within the KTH dataset, such as issues related to lighting, noise, and scaling. The proposed method extracted corner, blob, and ridge interest points, and all the challenges specific to the KTH dataset were thoroughly evaluated. Because of the large number of data involved, the classification method chosen was KNN, known for its effectiveness with big data. The proposed algorithm achieved an accuracy rate of 90% with this approach.

Additionally, Chakraborty and Mukhopadhyay [43] introduced a novel approach called heterogeneous recurrent spiking neural network (HRSNN), which uses unsupervised learning to classify spatiotemporal video activity recognition tasks. This approach was applied to various datasets, including KTH, in addition to event-based datasets. They achieved an accuracy rate of 94.32% specifically for the KTH dataset.

Moreover, Guo and Wang [44] proposed a model for recognizing human sports behavior by utilizing specific spatiotemporal features to extract and analyze information from large-scale video data. Their study improved the spatiotemporal deep belief network (DBN) and focused on enhancing the belief networks in DL using different pooling strategies. The proposed time–space DBN algorithm achieved an accuracy rate of 90% on the KTH dataset.

Finally, Liu et al. [45] introduced a learning algorithm that utilizes an evolutionary membrane algorithm to optimize the neural structure and hyperparameters of a liquid-state machine. To verify its effectiveness, the algorithm was tested on the MNIST and KTH datasets through simulation experiments. The algorithm achieved the best result of 86.3% accuracy on the KTH dataset.

Additionally, we conducted comparisons of the running time for each implemented model. As illustrated in Table 7, the single-frame CNN exhibited the longest training time, which is attributed to its construction from scratch and utilization of the traditional neural network approach. Conversely, the KNN model demonstrated the lowest time, although it yielded lower accuracy, 97%, compared with the higher accuracy of 98% achieved by VGG-16, which was achieved in a reasonable time due to its application of the transfer learning approach.

## 9. Conclusions

HAR is an important technology that has numerous applications across different domains. The development of DL algorithms, availability of large datasets, and parallel computing technologies have facilitated the development of accurate and real-time HAR systems. As the field continues to advance, it has the potential to revolutionize various domains, including health care, sports analysis, surveillance, and robotics.

In this study, we developed an imitation detection system using a CNN within the context of HAR, specifically targeting clapping imitation. We utilized transfer learning to build two models: a single-frame CNN and VGG-16. Performance evaluation using various metrics, including confusion matrix, accuracy, precision, recall, and F1, indicated that the VGG-16 model outperformed the single-frame CNN model.

One of the main challenges in HAR is the variability and complexity of human actions. Actions can differ significantly in terms of speed, duration, and style, posing difficulties in accurately recognizing and classifying all actions. Additionally, some actions may be ambiguous or resemble other similar actions, making it challenging for the system to identify them accurately. Moreover, the need for large number of labeled data to effectively train DL models is a challenge. Collecting and labeling data can be a time-consuming and expensive process, and obtaining a diverse and representative dataset that includes various actions and variations can be challenging. Real-world scenarios present further challenges for HAR systems, as they can be unpredictable and noisy. The system needs to handle changes in lighting conditions, occlusions, and other factors that can impact its performance. Additionally, robustness to individual differences is crucial, as different individuals may perform the same action in slightly different ways, requiring the system to recognize and adapt to these variations.

Future work for our research will address the aforementioned challenges and improve the system design to enable real-time analysis and visualization. In addition, enhancing the system’s accessibility online could enhance its usability. Furthermore, the imitation detection system could be expanded to recognize various movements beyond hand clapping, thereby extending its applicability and versatility. Importantly, a key future direction of our research is to advance the field and contribute to the development of more accurate and tailored solutions for behavior recognition in children with ASD. The focus on behavioral screening for children with ASD presents a valuable research point, and by integrating the characteristics of behavioral screening for children with ASD and further refining the methods proposed in this article, we could enhance the value and impact of our study.

## Figures and Tables

**Table 1:** Summary of HAR-related work.

Ref.	Year	Dataset	Accuracy	Algorithm	Type of Motion
[17]	2020	UCF Sports, UCF101, and KTH	96.3%—KTH	Combined (GRNN, GMM, and KF)	Full body
[18]	2021	UT-interaction	90.64%	Single CNN and VGG-16	Full body
[19]	2021	Pamap2	98%	LSTM RNN	Full body
[1]	2022	KTH, IXMAS, WVU, and Hollywood	99.9%—Hollywood	DL-based design	Full body
[20]	2021	LSP, LIP, and MPII	95%—LIP and MPII	SHN	Full body

**Table 2:** KTH dataset summary.

Number of classes	6 actions
Type of camera	Static camera with 25 fps frame rate
Types of movement	Walking, jogging, running, boxing, hand waving, and hand clapping
Number of clips	2391
Release date	2004

**Table 3:** Total loss vs. total accuracy for both models.

Single-Frame CNN	VGG-16
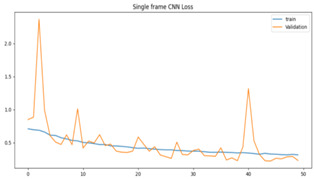 Single-frame CNN loss	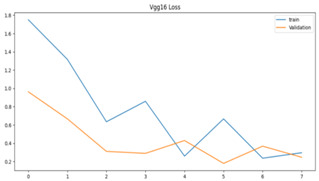 VGG-16 loss
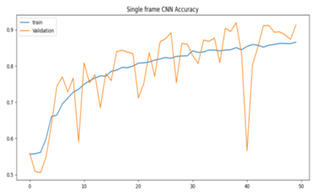 Single-frame CNN accuracy	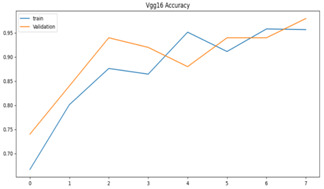 VGG-16 accuracy

**Table 4:** Confusion matrix.

Single-Frame CNN	VGG-16
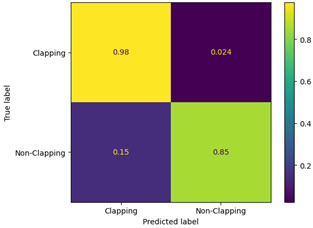	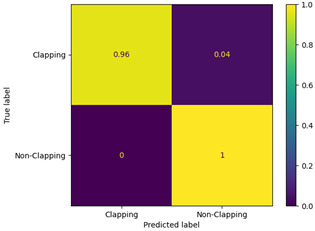

**Table 5:** Performance metrics.

Dataset	Algorithm	Accuracy (%)	Precision (%)	Recall (%)	F1 (%)
KTH	SVM	93	95	92	93
	RF	98	98	99	98
	KNN	97	99	96	97
	Single-frame CNN	91	97	85	90
	VGG-16	98	96	100	98

**Table 6:** Summary of comparison with similar approaches.

Ref.	Feature Extraction	Classifier	Accuracy
David and Abbas, 2019 [42]	Corner, blob, and ridge interest points	KNN	90%
Chakraborty and Mukhopadhyay, 2023 [43]	Spatiotemporal	HRSNN	94.32%
Guo and Wang, 2021 [44]	Spatiotemporal features	DBN	90%
Liu et al., 2022 [45]	LSM		86.3%
Our proposed model	VGG-16		98%

**Table 7:** Model running time comparison.

Algorithm	Running Time
SVM	12.8 s
RF	10.1 s
KNN	9.7 s
Single-frame CNN	936.7 s
VGG-16	20.6 s

## Data Availability

The KTH dataset used in this study is available at https://www.csc.kth.se/cvap/actions/ (accessed on 13 December 2023).
